# Perceptual uncertainty

**DOI:** 10.1371/journal.pbio.3000430

**Published:** 2019-08-27

**Authors:** Mathew E. Diamond

**Affiliations:** Cognitive Neuroscience, International School for Advanced Studies, Trieste, Italy

## Abstract

The number of the distinct tactile percepts exceeds the number of receptor types in the skin, signifying that perception cannot be explained by a one-to-one mapping from a single receptor channel to a corresponding percept. The abundance of touch experiences results from multiplexing (the coexistence of multiple codes within a single channel, increasing the available information content of that channel) and from the mixture of receptor channels by divergence and convergence. When a neuronal representation emerges through the combination of receptor channels, perceptual uncertainty can occur—a perceptual judgment is affected by a stimulus feature that would be, ideally, excluded from the task. Though uncertainty seems at first glance to reflect nonoptimality in sensory processing, it is actually a consequence of efficient coding mechanisms that exploit prior knowledge about objects that are touched. Studies that analyze how perceptual judgments are “fooled” by variations in sensory input can reveal the neuronal mechanisms underlying the tactile experience.

## Abstract 2

Perceptual uncertainty in sense of touch occurs when a judgment is affected by a stimulus feature that is irrelevant to the ongoing task. This Primer explores how studies that analyze how perceptual judgments are “fooled” by variations in sensory input can reveal the neuronal mechanisms underlying the tactile experience.

Nobody likes confusion. In politics, social policy, and many other fields of human activity, we prefer unequivocal answers. When the data do not support clear conclusions, we show a tendency to simplify the available knowledge to fit our scheme.

Our attempt to understand sensory systems—the receptors and central processing pathways that make us experience the external world—is no exception: we would prefer clean, circumscribed brain coding mechanisms for sensation. The “labeled lines” framework, whereby every form of sensation has its own dedicated processing system, originated with Aristotle; kindergarteners still learn his depiction of the five senses—seeing, hearing, touching, smelling, and tasting. Although it is a helpful scheme for youngsters, neuroscientists investigating the brain mechanisms of sensation and perception realize that a set of five labeled lines cannot come close to explaining our experience of the world.

One reason is that a single sensory modality is actually supported by several different sorts of receptors. Feelings arising from the interaction between the skin and an external object originate in an array of receptor structures, each of which has been identified by its specific “best stimulus” [1]. In the fingertip, four discrete receptor types are pertinent to the present line of argument (Fig 1A). The free nerve ending (FNE) is itself a family of chemoreceptors excited by a variety of different events—cooling and warming, noxious pinch or crush, chemical burning, and agents that itch or tickle [2]. Rapidly adapting type I receptors (RA1s), associated with the Meissner corpuscles, are excited by the onset and offset of light pressure at low frequency (up to some tens of hertz, usually as a vibration). Sensory fibers originating in the Pacinian corpuscle (PC) are excited by high-frequency compression and release, often in the form of vibration (with peak sensitivity at around 250 Hz). Slowly adapting type I receptors (SA1s), associated with Merkel discs, are excited by light pressure applied to the skin. We refer to these receptors and their afferent pathways as “primary” channels.

**Fig 1:**
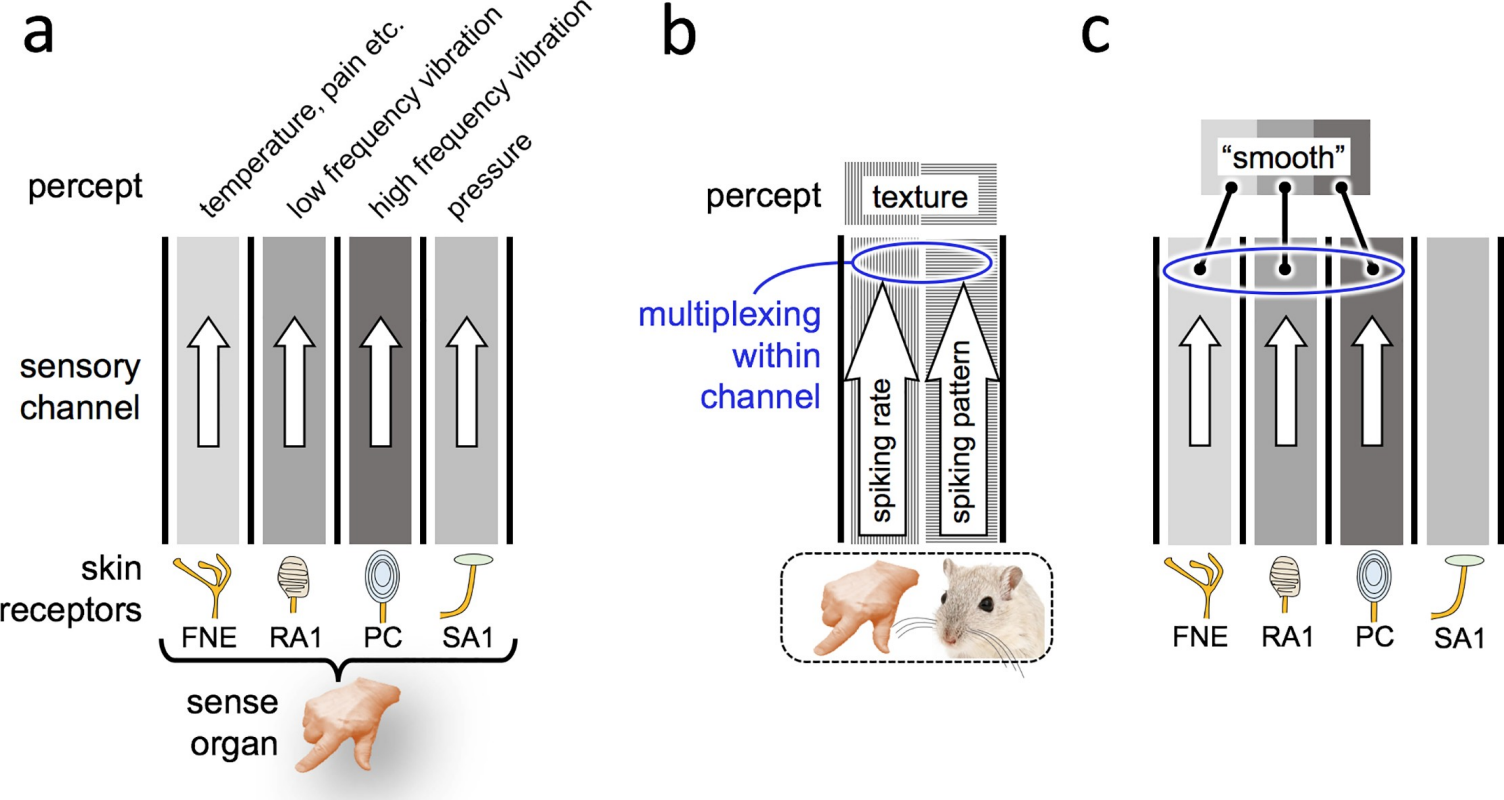
Percepts arising from somatosensory channels. (a) Four types of receptors in the skin of the fingertip and the percepts associated with their corresponding “best stimuli.” (b) For the primate fingertip and the rodent whiskers, multiplexing of spiking rate and spiking pattern codes gives rise to a more accurate texture percept. (c) Convergence of multiple channels can support a more complex percept, such as smoothness. FNE, free nerve ending; PC, Pacinian corpuscle; RA1, rapidly adapting type I receptor; SA1, slowly adapting type I receptor.

The identification of these channels and their corresponding best stimuli constitutes a necessary step toward solving the puzzle of skin sensing, but a major problem remains: the number of identifiable perceptual experiences is much larger than the number of primary channels. This numerical mismatch signifies that there cannot be a one-to-one correspondence between receptor type and percept. If we cast the skin percepts in feature dimensions, these dimensions will include surface microstructure (from rough to smooth), temperature (from warming to cooling), compliance (from soft to stiff), pointedness (from sharp to blunt), pressure (from light to strong), vibration frequency (from low to high), adhesiveness (from sticky to slippery), and moistness (from dry to wet). These feature dimensions only capture a small fraction of our tactile experience; even the perceptual space of texture is made up of dozens of dimensions. Furthermore, there are dynamic percepts, such as motion speed and direction. Note that whereas some of these dimensions can be mapped to receptor types (e.g., FNE expressing transient receptor potential (TRP) channels for thermal change [3]), most of them cannot.

What are the neuronal coding strategies that allow the number of perceptual dimensions to be much larger than the number of primary channels? How does the nervous system transmit and generate the largest possible quantity of information from channels that are small in number (in both the number of types of receptors and the number of receptors of each type that can be packed into a square centimeter of skin)? Here, we highlight two mechanisms for maximally exploiting a limited signal bandwidth.

The first is multiplexing, wherein multiple neuronal codes overlap within a single pathway. For instance, somatosensory pathways in rats [4,5] carry signals by two intermixed codes, spiking rate and temporal patterns of spikes (Fig 1B). The rate refers to the count of action potentials within some window that is at least a few times greater than the average interspike interval, typically in the neighborhood of 100 ms. Pattern refers to the temporal sequence of action potentials, considering time bins on the order of 5–10 ms or even less. When rats palpate a surface with their whiskers, somatosensory cortical neurons carry texture information both by the spike count in each touch and by the temporal configuration of those spikes [6]. The two multiplexed codes both contribute to the rat’s judgment of texture on each trial [7,8]. Likewise, when textured surfaces are presented to the primate fingertip, afferent fibers and somatosensory cortical neurons carry stimulus information both by the spike count and by the temporal configuration of those spikes [9,10]. Multiplexing makes a sensory code richer and more informative, expanding the quantity of distinct messages that can be carried. It reduces the ambiguity in our percept of what is being contacted, since two stimuli that evoke similar firing rates may be distinguished by a difference in firing pattern.

The second coding strategy is based on the divergence and convergence among primary channels. It can be easily appreciated that if each primary channel diverges to become a component of a larger set of percepts, the number of potential percepts grows in relation to the number of potential combinations. A distinctive feeling may derive from a specific combination of signals across multiple channels. For example, though there is no primary channel uniquely tasked with reporting that the hand is being dipped into a cool liquid, a distinct feeling of “wetness” emerges nonetheless from features like heat conduction, uniformity of spatial activation, and so on. Perceptual processing likely adopts a Bayesian algorithm to optimize the knowledge conveyed by convergence and divergence. At room temperature, highly smooth surfaces like Teflon usually feel cool because they rapidly conduct heat away from the fingertip. The fact that smooth surfaces typically feel cool can be inverted to surmise that a cool surface is likely to be smooth; in a Bayesian formulation, the probability that a surface is smooth conditional on it feeling cool is positively correlated with the probability that a surface feels cool conditional on it being smooth. Thus, in many situations, coolness amplifies the feeling of smoothness (Fig 1C).

Although the convergence and divergence of primary channels creates a broader set of available percepts, the same mechanism generates a coding conundrum that sometimes culminates in perceptual uncertainty. Perceptual confounds come about when a single feature contributes to multiple percepts. The case of surface coolness helps illustrate the point. If cooling boosts the percept of smoothness, then by reducing an object’s temperature our sensory system can be “fooled” into judging its surface as smoother than the skin mechanoreceptors (pressure, vibration, stretch, etc.) by themselves would encode it as being. It is interesting that in physics there also exist situations wherein one variable cannot be precisely measured unless another is known, such as the time–energy uncertainty [11].

In a paper published in this issue of *PLOS Biology*, Delhaye and colleagues [12] bring to light an additional case of somatosensory perceptual uncertainty; here, the stimulus of interest is motion across the skin. Tactile direction and speed are among the most critical sensory signals for survival. Feeling slip across the skin is crucial in applying the right grip force. In primates, motion acuity is high: blindfolded humans can distinguish the direction [13] and speed [14] at which objects move across the skin.

The investigators obtained from blindfolded human subjects perceptual judgments of speed as a variety of textured surfaces (including corduroy, stretch denim, nylon, mixed dots/grating, faux croc skin) were scanned across their stationary fingertip using a circular drum at speeds ranging from 20 mm/s to 120 mm/s in steps of 20 mm/s. Subjects rated the perceived speed of each moving surface using a numerical scale of their own choosing. Although all surfaces were perceived as moving faster as speed increased (as expected), some surfaces, at a given speed, were systematically perceived as moving faster than others. Nylon was felt as moving faster than corduroy, for instance. Furthermore, some textures yielded shallower speed functions than others, meaning that as real speed increased, subjective speed increased less. Speed of translation across the skin thus could not be judged in an absolute scale but was confounded by texture.

The second set of experiments attempted to understand the properties of neuronal coding that might explain the interaction between speed and texture. The investigators recorded the activity of SA1, RA1, and PC fibers from the nerve of anesthetized monkeys as textures were scanned at different speeds across the fingertips. Although the number of studied fibers was small, the data set was sufficient to determine that the firing rate of all afferent types increased with speed but to different degrees: PC responses were more dependent on speed than were the other types. Importantly, PC responses also depended on texture and could thus account for differences in the texture-dependent perceptual sensitivity to speed. That is, considering surfaces in pairwise comparisons, the texture that evoked a stronger response in PC fibers was systematically perceived as moving faster. In somatosensory cortex, neuronal correlates of the perceptual confound also emerged, though the findings were complex; speed and texture codes were entangled and larger-scale population decoding concomitant with execution of the psychophysical task is needed to support a better understanding.

The connection between PC firing, speed, and texture is explained by the skin vibrations evoked by movement of the surfaces. Delhaye and colleagues [12] measured the high-frequency vibratory power in the finger pad by laser-Doppler vibrometry and found that power increased with speed and varied across textures. In conclusion, the strength of vibrations in the skin grows in relation to surface speed, evoking progressively larger responses in PC fibers and leading to a speed estimation mechanism. But the PC channel is also excited differentially according to the texture applied. The outcome is perceptual uncertainty (Fig 2).

**Fig 2:**
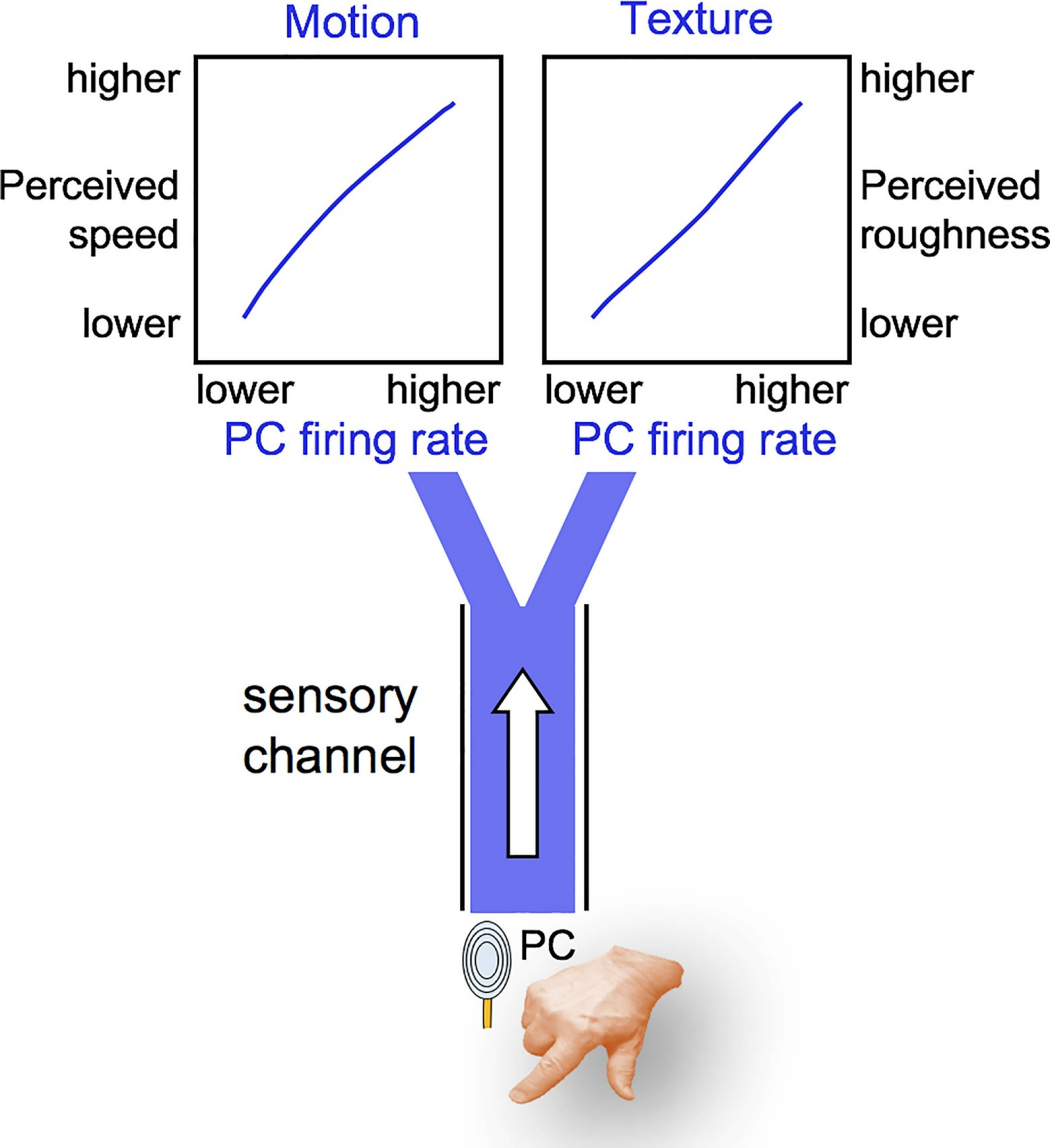
How texture fools the speed percept. Signals from the PC receptors diverge and contribute to two percepts, motion and texture. A higher PC firing rate encodes greater speed across the skin, but it also encodes texture properties. Because the decoding stage of the pathway cannot be certain whether a given level of PC firing arises from speed or texture, a confound emerges. PC, Pacinian corpuscle.

If our sensory–perceptual processing systems are replete with uncertain signals, how does the brain accurately recognize the things around us? One strategy is cross-modal sensory integration. It is common to deploy more than one sensory system to interpret real-world events, and the combination of modalities may resolve the uncertainty inherent to a single modality (Fig 3). When rats were trained to judge the orientation of a raised, black and white grating by visual, tactile, and visual–tactile inspection, performance in the visual–tactile condition was better than that predicted by optimal linear combination of visual and tactile signals, indicating synergy between sensory channels [15]. Posterior parietal cortex—situated between visual and somatosensory cortex—was examined as a potential site for the visual–tactile convergence, and a linear classifier of neuronal population activity replicated the behavioral findings. Taken together, these findings suggest that the synergy between sensory modalities, perhaps occurring within posterior parietal cortex, reduces the perceptual uncertainty inherent to a single sensory system. It is interesting to consider that visual signals under some conditions might solve the texture–speed conundrum reported by Delhaye and colleagues [12]: inspection of the moving object, together with the tactile signals, would reduce uncertainty.

**Fig 3:**
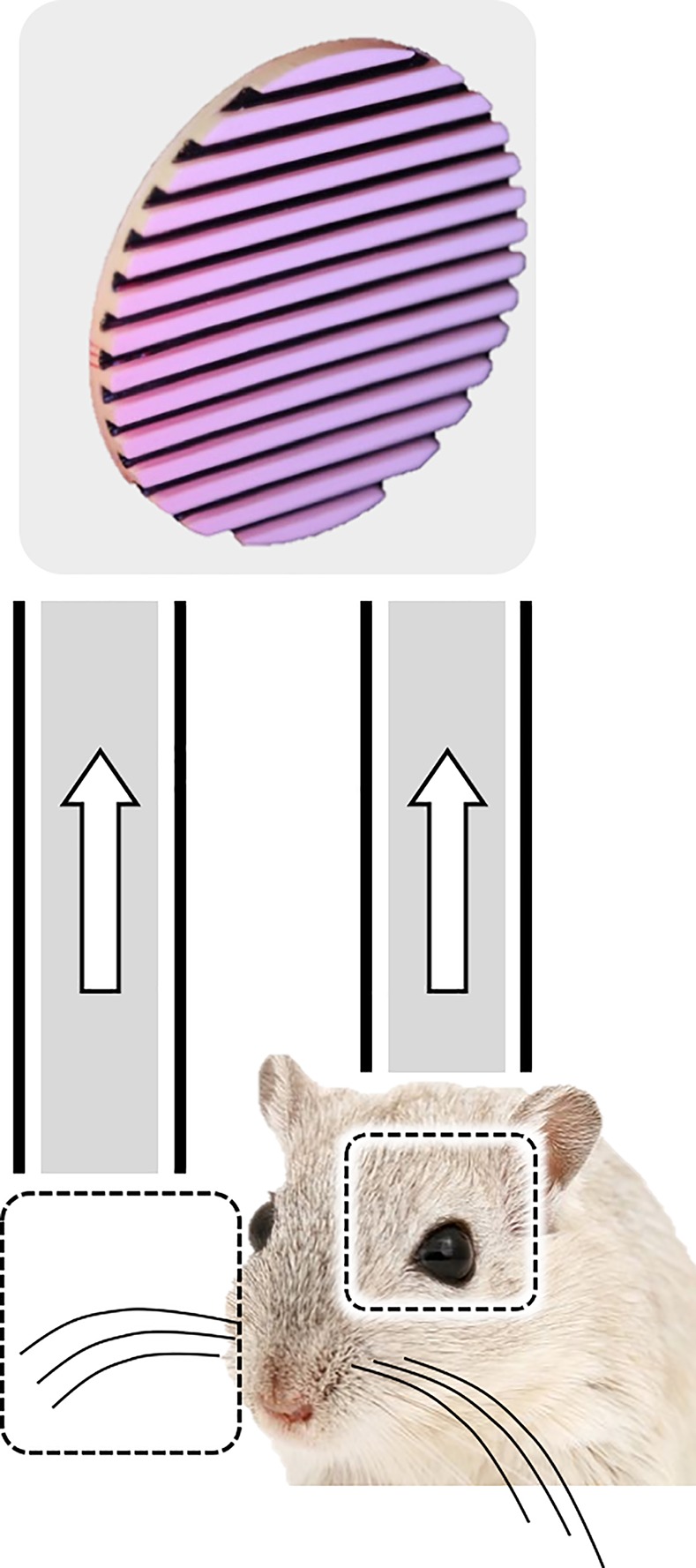
Reduction of perceptual uncertainty. Cross-modal sensory integration. To estimate the orientation of the bars when the signal within a single sensory channel is uncertain, tactile and visual sensory channels are combined in a supralinear manner.

A second uncertainty-reducing strategy is to employ high-level contextual knowledge, information that does not come directly through the senses. Spicy food feels “hot” on the tongue because it excites the same pathway—the C-fiber population expressing TRP channels—otherwise used to signal burning by high temperature [16]. But the consumer of a fine Indonesian sambal does not attribute the burning sensation to temperature, because the context provides a better explanation for the experience.

Perceptual uncertainty, more than a source of frustration to neuroscientists, can be instrumental in uncovering sensory processing mechanisms. Rats are unable to form distinct percepts of the amplitude (*A*) and frequency (*f*) of a sinusoidal vibration received through the whiskers; instead, they perceive the product, *Af*. In other words, they can distinguish vibrations 1 and 2 only if *A1f1* is not equal to *A2f2* [17]. This perceptual uncertainty (*A* cannot be known unless *f* is known, and vice versa) informs us that vibration mean speed—the physical feature corresponding to *Af*—is extracted by the sensory system and converted to an intensity percept, consistent with evidence from vibration coding by cortical neurons [18,19].

Furthermore, psychophysical experiments that reveal the interaction between two percepts are a clue to some common component in the underlying neuronal representation of both percepts. In humans and in rats, the duration of a vibration systematically shifts its perceived intensity [20], and the intensity of a vibration likewise shifts its perceived duration [21]. This perceptual uncertainty (duration cannot be known unless intensity is known, and vice versa) allows us to formulate the construction of the intensity percept and the construction of the time percept within a unified framework—a common drive within the somatosensory representation leading to two very different forms of experience [21]. In sum, confusion, paradoxically, can help sort out the neuronal bases of touch perception.

## Abbreviations

A: amplitude of a sinusoidal vibration
f: frequency of a sinusoidal vibration
FNE: free nerve ending
PC: Pacinian corpuscle
RA1: rapidly adapting type I receptor
SA1: slowly adapting type I receptor
TRP: transient receptor potential
